# Dibenzyl 3,3′-diethyl-4,4′-dimethyl-2,2′-methylenebis(pyrrole-5-carboxylate)

**DOI:** 10.1107/S1600536810004459

**Published:** 2010-02-10

**Authors:** Hee-Joon Kim

**Affiliations:** a Department of Applied Chemistry, Kumoh National Institute of Technology, 1 Yangho-dong, Gumi 730-701, Republic of Korea

## Abstract

In the title compound, C_31_H_34_N_2_O_4_, the two pyrrole rings are bent around the central methyl­ene C atom, making a dihedral angle of 64.83 (7)°. In the crystal, mol­ecules are linked into dimers *via* N—H⋯O=C hydrogen bonds. These dimers are packed through π⋯π inter­actions between neighboring pyrrole rings with a separation between the mean planes of symmetry-related pyrrole rings of 3.61 (2) Å and a centroid–centroid distance of 4.33 Å. Parallel phenyl groups in neighboring dimers also exhibit efficient π⋯π inter­actions, characterized by an inter­plane separation of 3.378 (8) Å and a centroid–centroid distance of 3.97 Å.

## Related literature

For the preparation of the title compound, see: Twyman & Sanders (1999 ▶). For related structures, see: Bonnett *et al.* (1972 ▶); Senge (2005 ▶); Vega *et al.* (2003 ▶). For the use of dipyrrylmethanes in organic synthesis, see: Chen *et al.* (2000 ▶) and references cited therein; Jasat & Dolphin (1997 ▶); Shanmugathasan *et al.* (2000 ▶). 
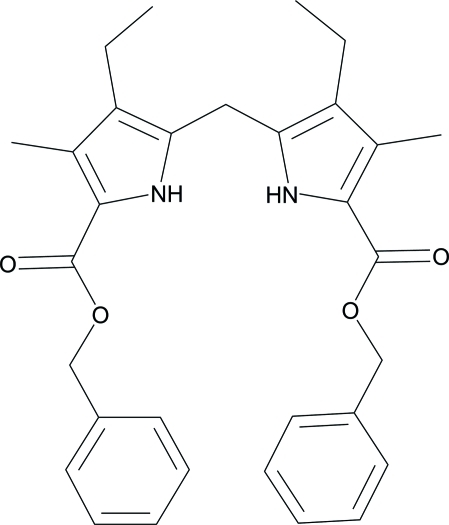


## Experimental

### 

#### Crystal data

C_31_H_34_N_2_O_4_
*M* *_r_* = 498.60
Monoclinic, 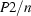

*a* = 14.2002 (9) Å
*b* = 7.9220 (5) Å
*c* = 25.0939 (16) Å
β = 104.373 (3)°
*V* = 2734.6 (3) Å^3^
*Z* = 4
Mo *K*α radiation
μ = 0.08 mm^−1^
*T* = 162 K
0.35 × 0.32 × 0.08 mm

#### Data collection

Bruker SMART APEX CCD diffractometer
Absorption correction: multi-scan (*SADABS*; Bruker, 1998 ▶) *T*_min_ = 0.972, *T*_max_ = 0.993
37570 measured reflections
6304 independent reflections
4371 reflections with *I* > 2σ(*I*)
*R*_int_ = 0.059

#### Refinement

*R*[*F* ^2^ > 2σ(*F* ^2^)] = 0.049
*wR*(*F* ^2^) = 0.136
*S* = 1.05
6304 reflections
346 parameters
H atoms treated by a mixture of independent and constrained refinement
Δρ_max_ = 0.29 e Å^−3^
Δρ_min_ = −0.26 e Å^−3^

### 

Data collection: *SMART* (Bruker, 1998 ▶); cell refinement: *SAINT* (Bruker, 1998 ▶); data reduction: *SAINT*; program(s) used to solve structure: *SHELXTL* (Sheldrick, 2008 ▶); program(s) used to refine structure: *SHELXTL*; molecular graphics: *SHELXTL*; software used to prepare material for publication: *SHELXTL*.

## Supplementary Material

Crystal structure: contains datablocks I, globle. DOI: 10.1107/S1600536810004459/bh2271sup1.cif

Structure factors: contains datablocks I. DOI: 10.1107/S1600536810004459/bh2271Isup2.hkl

Additional supplementary materials:  crystallographic information; 3D view; checkCIF report

## Figures and Tables

**Table 1:** Hydrogen-bond geometry (Å, °)

*D*—H⋯*A*	*D*—H	H⋯*A*	*D*⋯*A*	*D*—H⋯*A*
N1—H1⋯O3^i^	0.87 (2)	2.09 (2)	2.9334 (18)	163.4 (18)
N2—H2⋯O3^i^	0.89 (2)	2.00 (2)	2.8610 (17)	162.8 (17)

Symmetry code: (i) 
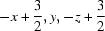
.

**Fig. 1.**
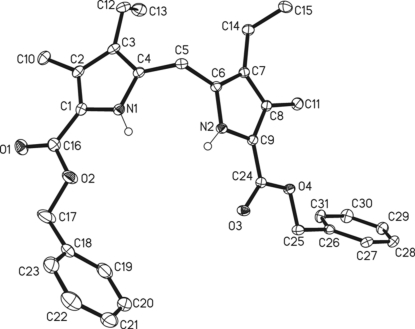
The molecular structure of the title compound showing 30 % probability displacement ellipsoids. Hydrogen atoms except for the N—H groups are omitted for clarity.

**Fig. 2.**
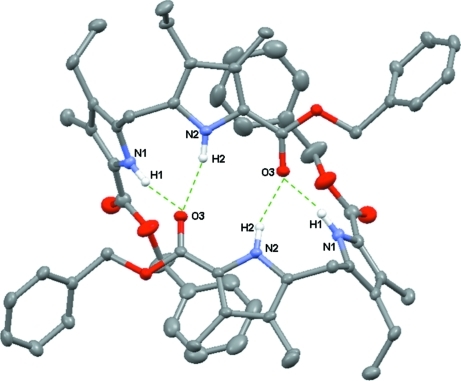
Hydrogen bonded dimer in the crystal of the title compound.

**Fig. 3.**
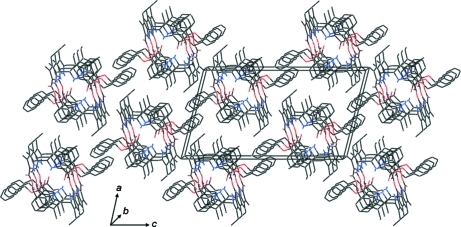
Packing diagram for the hydrogen bonded dimers of the title compound in the crystal lattice.

## supplementary crystallographic information

### Comment

Dipyrrylmethanes have been widely used as versatile precursors in the synthesis of porphyrins (Shanmugathasan *et al.*, 2000), related polypyrrolic macrocycles (Jasat *et al.*, 1997) and pigments (Chen *et al.*, 2000). For the synthesis of novel porphyrins, the title compound was prepared and its crystal structure determined. Related structure of dipyrrylmethanes derivatives were previously reported (Bonnett *et al.*, 1972; Senge *et al.*, 2005; Vega *et al.*, 2003).

The molecular structure of the title compound is shown in Figure 1. The two pyrrole rings are bent with the dihedral angle of 64.83 (7)° around the attached methylene carbon atom C5. As shown in Figure 2, the molecules are linked by paired N—H···O=C hydrogen bonds into dimers in the crystal lattice. The structural parameters for the intermolecular hydrogen bonds resulting in the formation of dimers are given in Table 1. These dimeric units are packed through π···π interactions between neighboring pyrrole rings as well as neighboring phenyl rings as shown in Figure 3. The interplane and a centroid-to-centroid separations between the parallel pyrrole groups are 3.61 (2) and 4.33 Å, respectively. The parallel phenyl groups in neighboring dimers in the crystal also exhibit efficient π···π interactions: interplane separation of 3.378 (8) Å, and centroid-to-centroid distance of 3.973 Å.

### Experimental

The title compound was prepared according to the reported procedure (Twyman *et al.*, 1999). Crystals suitable for X-ray crystallographic work were grown by slow vapor diffusion of *n*-hexane into a toluene solution.

### Refinement

All non-H atoms were refined anisotropically. C-bonded H atoms were placed in geometrically ideal positions and refined as riding to their parent C atoms with C—H bond lengths fixed to 0.99 (methylene) or 0.98 Å (methyl). Displacement parameters were computed as *U*_iso_(H) = 1.2*U*_eq_(carrier C) for methylene groups and *U*_iso_(H) = 1.5*U*_eq_(carrier C) for methyl groups. Atoms H1 and H2, bonded to N1 and N2, were found in a difference map and refined freely (coordinates and displacement parameters).

### Crystal data

**Table:**

C_31_H_34_N_2_O_4_	*F*(000) = 1064
*M**_r_* = 498.60	*D*_x_ = 1.211 Mg m^−^^3^
Monoclinic, *P*2/*n*	Mo *K*α radiation, λ = 0.71073 Å
Hall symbol: -P 2yac	Cell parameters from 9928 reflections
*a* = 14.2002 (9) Å	θ = 2.6–24.4°
*b* = 7.9220 (5) Å	µ = 0.08 mm^−^^1^
*c* = 25.0939 (16) Å	*T* = 162 K
β = 104.373 (3)°	Plate, colourless
*V* = 2734.6 (3) Å^3^	0.35 × 0.32 × 0.08 mm
*Z* = 4	

### Data collection

**Table:**

Bruker SMART APEX CCD diffractometer	6304 independent reflections
Radiation source: fine-focus sealed tube	4371 reflections with *I* > 2σ(*I*)
graphite	*R*_int_ = 0.059
φ and ω scans	θ_max_ = 27.6°, θ_min_ = 1.5°
Absorption correction: multi-scan (*SADABS*; Bruker, 1998)	*h* = −18→18
*T*_min_ = 0.972, *T*_max_ = 0.993	*k* = −10→10
37570 measured reflections	*l* = −31→32

### Refinement

**Table:**

Refinement on *F*^2^	Primary atom site location: structure-invariant direct methods
Least-squares matrix: full	Secondary atom site location: difference Fourier map
*R*[*F*^2^ > 2σ(*F*^2^)] = 0.049	Hydrogen site location: inferred from neighbouring sites
*wR*(*F*^2^) = 0.136	H atoms treated by a mixture of independent and constrained refinement
*S* = 1.05	*w* = 1/[σ^2^(*F*_o_^2^) + (0.0505*P*)^2^ + 0.953*P*] where *P* = (*F*_o_^2^ + 2*F*_c_^2^)/3
6304 reflections	(Δ/σ)_max_ = 0.001
346 parameters	Δρ_max_ = 0.29 e Å^−^^3^
0 restraints	Δρ_min_ = −0.26 e Å^−^^3^
0 constraints	

### Fractional atomic coordinates and isotropic or equivalent isotropic displacement parameters (Å^2^)

**Table:**

	*x*	*y*	*z*	*U*_iso_*/*U*_eq_	
O1	0.84641 (10)	0.85654 (17)	0.91775 (6)	0.0528 (4)	
O2	0.75643 (10)	0.65074 (17)	0.86750 (7)	0.0603 (4)	
O3	0.76635 (7)	0.24675 (15)	0.68188 (4)	0.0342 (3)	
O4	0.87580 (7)	0.18152 (15)	0.63406 (4)	0.0334 (3)	
N1	0.90073 (10)	0.43117 (18)	0.88502 (5)	0.0310 (3)	
H1	0.8449 (15)	0.396 (3)	0.8648 (8)	0.047 (6)*	
N2	0.90338 (9)	0.17664 (17)	0.77990 (5)	0.0285 (3)	
H2	0.8449 (14)	0.204 (2)	0.7845 (7)	0.045 (5)*	
C1	0.91713 (12)	0.5906 (2)	0.90767 (7)	0.0339 (4)	
C2	1.01355 (12)	0.5990 (2)	0.93634 (6)	0.0348 (4)	
C3	1.05588 (12)	0.4413 (2)	0.93042 (6)	0.0327 (4)	
C4	0.98377 (11)	0.3399 (2)	0.89853 (6)	0.0295 (3)	
C5	0.98552 (12)	0.1610 (2)	0.87970 (6)	0.0319 (4)	
H5A	0.9283	0.1010	0.8863	0.038*	
H5B	1.0445	0.1051	0.9021	0.038*	
C6	0.98473 (11)	0.1445 (2)	0.82008 (6)	0.0294 (3)	
C7	1.05896 (11)	0.1041 (2)	0.79533 (7)	0.0315 (4)	
C8	1.01998 (11)	0.1126 (2)	0.73787 (7)	0.0310 (4)	
C9	0.92347 (11)	0.1592 (2)	0.72921 (6)	0.0287 (3)	
C10	1.06422 (14)	0.7464 (2)	0.96891 (8)	0.0461 (5)	
H10A	1.0161	0.8318	0.9723	0.069*	
H10B	1.1105	0.7954	0.9501	0.069*	
H10C	1.0991	0.7081	1.0056	0.069*	
C11	1.07570 (12)	0.0804 (3)	0.69528 (7)	0.0426 (4)	
H11A	1.0525	0.1566	0.6640	0.064*	
H11B	1.0659	−0.0369	0.6827	0.064*	
H11C	1.1451	0.1004	0.7113	0.064*	
C12	1.16037 (12)	0.3931 (3)	0.95415 (7)	0.0422 (4)	
H12A	1.1652	0.2685	0.9563	0.051*	
H12B	1.1815	0.4379	0.9921	0.051*	
C13	1.22892 (14)	0.4582 (3)	0.92102 (9)	0.0538 (5)	
H13A	1.2952	0.4199	0.9380	0.081*	
H13B	1.2273	0.5818	0.9203	0.081*	
H13C	1.2085	0.4148	0.8833	0.081*	
C14	1.16166 (11)	0.0618 (2)	0.82435 (7)	0.0399 (4)	
H14A	1.1742	0.1008	0.8630	0.048*	
H14B	1.2061	0.1243	0.8067	0.048*	
C15	1.18485 (14)	−0.1252 (3)	0.82408 (9)	0.0533 (5)	
H15A	1.2535	−0.1434	0.8425	0.080*	
H15B	1.1721	−0.1653	0.7860	0.080*	
H15C	1.1440	−0.1875	0.8435	0.080*	
C16	0.84033 (13)	0.7142 (2)	0.89944 (8)	0.0416 (4)	
C17	0.67700 (17)	0.7704 (3)	0.85195 (13)	0.0862 (9)	
H17A	0.6956	0.8644	0.8306	0.103*	
H17B	0.6614	0.8179	0.8852	0.103*	
C18	0.59147 (15)	0.6804 (3)	0.81813 (9)	0.0532 (5)	
C19	0.58054 (18)	0.6486 (3)	0.76239 (10)	0.0682 (7)	
H19A	0.6301	0.6828	0.7453	0.082*	
C20	0.4995 (2)	0.5688 (3)	0.73183 (10)	0.0717 (7)	
H20A	0.4937	0.5482	0.6939	0.086*	
C21	0.4286 (2)	0.5196 (3)	0.75463 (12)	0.0751 (8)	
H21A	0.3728	0.4646	0.7328	0.090*	
C22	0.43577 (19)	0.5477 (3)	0.80892 (13)	0.0766 (8)	
H22A	0.3850	0.5135	0.8250	0.092*	
C23	0.51800 (19)	0.6269 (3)	0.84062 (10)	0.0637 (6)	
H23A	0.5234	0.6445	0.8787	0.076*	
C24	0.84815 (11)	0.1997 (2)	0.68106 (6)	0.0276 (3)	
C25	0.80269 (12)	0.2219 (2)	0.58423 (6)	0.0359 (4)	
H25A	0.7837	0.3421	0.5844	0.043*	
H25B	0.7441	0.1514	0.5814	0.043*	
C26	0.84675 (12)	0.1871 (2)	0.53687 (6)	0.0324 (4)	
C27	0.81372 (12)	0.0548 (2)	0.50133 (7)	0.0405 (4)	
H27A	0.7629	−0.0156	0.5069	0.049*	
C28	0.85450 (14)	0.0244 (3)	0.45761 (7)	0.0478 (5)	
H28A	0.8312	−0.0662	0.4331	0.057*	
C29	0.92838 (15)	0.1244 (3)	0.44945 (7)	0.0490 (5)	
H29A	0.9560	0.1032	0.4193	0.059*	
C30	0.96260 (15)	0.2558 (3)	0.48502 (8)	0.0496 (5)	
H30A	1.0142	0.3247	0.4796	0.060*	
C31	0.92170 (14)	0.2872 (2)	0.52860 (7)	0.0414 (4)	
H31A	0.9452	0.3780	0.5530	0.050*	

### Atomic displacement parameters (Å^2^)

**Table:**

	*U*^11^	*U*^22^	*U*^33^	*U*^12^	*U*^13^	*U*^23^
O1	0.0556 (8)	0.0344 (8)	0.0696 (9)	−0.0040 (6)	0.0177 (7)	−0.0095 (7)
O2	0.0426 (8)	0.0386 (8)	0.0881 (11)	0.0093 (6)	−0.0059 (7)	−0.0128 (7)
O3	0.0246 (6)	0.0458 (7)	0.0335 (6)	0.0038 (5)	0.0095 (5)	0.0045 (5)
O4	0.0264 (6)	0.0474 (7)	0.0267 (5)	0.0018 (5)	0.0072 (5)	−0.0004 (5)
N1	0.0286 (7)	0.0331 (8)	0.0300 (7)	−0.0034 (6)	0.0047 (6)	−0.0023 (6)
N2	0.0198 (6)	0.0365 (8)	0.0293 (7)	0.0011 (6)	0.0067 (5)	−0.0012 (6)
C1	0.0390 (9)	0.0325 (9)	0.0308 (8)	−0.0042 (7)	0.0098 (7)	−0.0021 (7)
C2	0.0396 (9)	0.0388 (10)	0.0255 (8)	−0.0079 (8)	0.0073 (7)	0.0008 (7)
C3	0.0335 (9)	0.0401 (10)	0.0227 (7)	−0.0062 (7)	0.0035 (6)	0.0036 (7)
C4	0.0287 (8)	0.0353 (9)	0.0241 (7)	−0.0025 (7)	0.0061 (6)	0.0021 (7)
C5	0.0295 (8)	0.0349 (9)	0.0297 (8)	0.0011 (7)	0.0042 (7)	0.0040 (7)
C6	0.0240 (7)	0.0305 (9)	0.0317 (8)	−0.0014 (6)	0.0035 (6)	−0.0017 (7)
C7	0.0229 (8)	0.0337 (9)	0.0365 (9)	−0.0017 (7)	0.0046 (6)	−0.0067 (7)
C8	0.0220 (7)	0.0355 (9)	0.0355 (8)	−0.0026 (7)	0.0073 (6)	−0.0065 (7)
C9	0.0238 (7)	0.0340 (9)	0.0288 (8)	−0.0018 (6)	0.0074 (6)	−0.0033 (7)
C10	0.0522 (11)	0.0449 (11)	0.0387 (10)	−0.0128 (9)	0.0062 (8)	−0.0066 (9)
C11	0.0289 (9)	0.0586 (12)	0.0426 (10)	0.0007 (8)	0.0137 (7)	−0.0106 (9)
C12	0.0365 (10)	0.0486 (11)	0.0344 (9)	−0.0041 (8)	−0.0044 (7)	0.0032 (8)
C13	0.0353 (10)	0.0589 (13)	0.0647 (13)	−0.0043 (9)	0.0075 (9)	0.0014 (11)
C14	0.0229 (8)	0.0482 (11)	0.0455 (10)	0.0012 (7)	0.0026 (7)	−0.0091 (9)
C15	0.0381 (10)	0.0506 (12)	0.0624 (13)	0.0104 (9)	−0.0042 (9)	−0.0059 (10)
C16	0.0437 (10)	0.0353 (10)	0.0461 (10)	−0.0035 (8)	0.0115 (8)	−0.0010 (8)
C17	0.0564 (14)	0.0475 (14)	0.133 (2)	0.0215 (11)	−0.0170 (15)	−0.0224 (15)
C18	0.0521 (12)	0.0439 (12)	0.0589 (13)	0.0229 (10)	0.0051 (10)	−0.0037 (10)
C19	0.0650 (15)	0.0705 (16)	0.0724 (16)	0.0287 (13)	0.0233 (13)	0.0057 (13)
C20	0.0748 (17)	0.0749 (17)	0.0536 (13)	0.0352 (14)	−0.0066 (13)	−0.0125 (13)
C21	0.0650 (16)	0.0567 (15)	0.089 (2)	0.0198 (13)	−0.0085 (14)	−0.0120 (14)
C22	0.0667 (16)	0.0588 (16)	0.108 (2)	0.0098 (13)	0.0284 (16)	0.0136 (16)
C23	0.0788 (17)	0.0564 (14)	0.0533 (13)	0.0223 (13)	0.0112 (12)	0.0034 (11)
C24	0.0241 (7)	0.0294 (8)	0.0307 (8)	−0.0033 (6)	0.0096 (6)	−0.0001 (7)
C25	0.0302 (8)	0.0458 (10)	0.0304 (8)	0.0040 (7)	0.0053 (7)	0.0059 (8)
C26	0.0316 (8)	0.0368 (9)	0.0276 (8)	0.0038 (7)	0.0051 (7)	0.0050 (7)
C27	0.0331 (9)	0.0471 (11)	0.0364 (9)	−0.0033 (8)	−0.0005 (7)	−0.0010 (8)
C28	0.0486 (11)	0.0553 (12)	0.0332 (9)	0.0020 (10)	−0.0018 (8)	−0.0113 (9)
C29	0.0591 (12)	0.0605 (13)	0.0285 (9)	0.0076 (10)	0.0133 (8)	−0.0004 (9)
C30	0.0582 (12)	0.0535 (13)	0.0430 (10)	−0.0070 (10)	0.0236 (9)	0.0045 (10)
C31	0.0520 (11)	0.0392 (10)	0.0353 (9)	−0.0073 (8)	0.0154 (8)	−0.0030 (8)

### Geometric parameters (Å, °)

**Table:**

O1—C16	1.213 (2)	C13—H13A	0.9800
O2—C16	1.356 (2)	C13—H13B	0.9800
O2—C17	1.451 (2)	C13—H13C	0.9800
O3—C24	1.2249 (18)	C14—C15	1.518 (3)
O4—C24	1.3400 (18)	C14—H14A	0.9900
O4—C25	1.4490 (18)	C14—H14B	0.9900
N1—C4	1.352 (2)	C15—H15A	0.9800
N1—C1	1.381 (2)	C15—H15B	0.9800
N1—H1	0.87 (2)	C15—H15C	0.9800
N2—C6	1.356 (2)	C17—C18	1.481 (3)
N2—C9	1.3778 (19)	C17—H17A	0.9900
N2—H2	0.893 (19)	C17—H17B	0.9900
C1—C2	1.381 (2)	C18—C23	1.371 (3)
C1—C16	1.442 (3)	C18—C19	1.391 (3)
C2—C3	1.410 (3)	C19—C20	1.368 (4)
C2—C10	1.502 (2)	C19—H19A	0.9500
C3—C4	1.388 (2)	C20—C21	1.334 (4)
C3—C12	1.504 (2)	C20—H20A	0.9500
C4—C5	1.496 (2)	C21—C22	1.359 (4)
C5—C6	1.499 (2)	C21—H21A	0.9500
C5—H5A	0.9900	C22—C23	1.389 (4)
C5—H5B	0.9900	C22—H22A	0.9500
C6—C7	1.387 (2)	C23—H23A	0.9500
C7—C8	1.412 (2)	C25—C26	1.500 (2)
C7—C14	1.497 (2)	C25—H25A	0.9900
C8—C9	1.383 (2)	C25—H25B	0.9900
C8—C11	1.501 (2)	C26—C27	1.381 (2)
C9—C24	1.437 (2)	C26—C31	1.384 (2)
C10—H10A	0.9800	C27—C28	1.383 (3)
C10—H10B	0.9800	C27—H27A	0.9500
C10—H10C	0.9800	C28—C29	1.370 (3)
C11—H11A	0.9800	C28—H28A	0.9500
C11—H11B	0.9800	C29—C30	1.379 (3)
C11—H11C	0.9800	C29—H29A	0.9500
C12—C13	1.519 (3)	C30—C31	1.382 (2)
C12—H12A	0.9900	C30—H30A	0.9500
C12—H12B	0.9900	C31—H31A	0.9500
			
C16—O2—C17	115.28 (16)	C15—C14—H14A	108.8
C24—O4—C25	115.60 (12)	C7—C14—H14B	108.8
C4—N1—C1	109.76 (14)	C15—C14—H14B	108.8
C4—N1—H1	125.8 (13)	H14A—C14—H14B	107.7
C1—N1—H1	124.5 (13)	C14—C15—H15A	109.5
C6—N2—C9	109.53 (13)	C14—C15—H15B	109.5
C6—N2—H2	126.6 (12)	H15A—C15—H15B	109.5
C9—N2—H2	123.8 (12)	C14—C15—H15C	109.5
N1—C1—C2	107.62 (15)	H15A—C15—H15C	109.5
N1—C1—C16	121.18 (15)	H15B—C15—H15C	109.5
C2—C1—C16	131.20 (16)	O1—C16—O2	122.53 (17)
C1—C2—C3	107.20 (15)	O1—C16—C1	126.65 (18)
C1—C2—C10	126.87 (17)	O2—C16—C1	110.82 (16)
C3—C2—C10	125.92 (16)	O2—C17—C18	108.16 (17)
C4—C3—C2	107.49 (14)	O2—C17—H17A	110.1
C4—C3—C12	126.53 (16)	C18—C17—H17A	110.1
C2—C3—C12	125.98 (15)	O2—C17—H17B	110.1
N1—C4—C3	107.93 (15)	C18—C17—H17B	110.1
N1—C4—C5	120.59 (14)	H17A—C17—H17B	108.4
C3—C4—C5	131.48 (15)	C23—C18—C19	116.8 (2)
C4—C5—C6	113.68 (13)	C23—C18—C17	120.8 (2)
C4—C5—H5A	108.8	C19—C18—C17	122.3 (2)
C6—C5—H5A	108.8	C20—C19—C18	121.0 (2)
C4—C5—H5B	108.8	C20—C19—H19A	119.5
C6—C5—H5B	108.8	C18—C19—H19A	119.5
H5A—C5—H5B	107.7	C21—C20—C19	120.9 (2)
N2—C6—C7	108.19 (14)	C21—C20—H20A	119.6
N2—C6—C5	121.31 (13)	C19—C20—H20A	119.6
C7—C6—C5	130.45 (14)	C20—C21—C22	120.4 (3)
C6—C7—C8	107.30 (13)	C20—C21—H21A	119.8
C6—C7—C14	126.19 (15)	C22—C21—H21A	119.8
C8—C7—C14	126.51 (14)	C21—C22—C23	119.4 (3)
C9—C8—C7	107.16 (13)	C21—C22—H22A	120.3
C9—C8—C11	127.60 (15)	C23—C22—H22A	120.3
C7—C8—C11	125.22 (14)	C18—C23—C22	121.4 (2)
N2—C9—C8	107.80 (13)	C18—C23—H23A	119.3
N2—C9—C24	118.19 (13)	C22—C23—H23A	119.3
C8—C9—C24	133.88 (14)	O3—C24—O4	122.27 (14)
C2—C10—H10A	109.5	O3—C24—C9	124.37 (14)
C2—C10—H10B	109.5	O4—C24—C9	113.36 (13)
H10A—C10—H10B	109.5	O4—C25—C26	107.03 (13)
C2—C10—H10C	109.5	O4—C25—H25A	110.3
H10A—C10—H10C	109.5	C26—C25—H25A	110.3
H10B—C10—H10C	109.5	O4—C25—H25B	110.3
C8—C11—H11A	109.5	C26—C25—H25B	110.3
C8—C11—H11B	109.5	H25A—C25—H25B	108.6
H11A—C11—H11B	109.5	C27—C26—C31	119.28 (16)
C8—C11—H11C	109.5	C27—C26—C25	120.65 (16)
H11A—C11—H11C	109.5	C31—C26—C25	120.07 (16)
H11B—C11—H11C	109.5	C26—C27—C28	120.15 (17)
C3—C12—C13	113.60 (15)	C26—C27—H27A	119.9
C3—C12—H12A	108.8	C28—C27—H27A	119.9
C13—C12—H12A	108.8	C29—C28—C27	120.34 (18)
C3—C12—H12B	108.8	C29—C28—H28A	119.8
C13—C12—H12B	108.8	C27—C28—H28A	119.8
H12A—C12—H12B	107.7	C28—C29—C30	119.98 (17)
C12—C13—H13A	109.5	C28—C29—H29A	120.0
C12—C13—H13B	109.5	C30—C29—H29A	120.0
H13A—C13—H13B	109.5	C29—C30—C31	119.88 (18)
C12—C13—H13C	109.5	C29—C30—H30A	120.1
H13A—C13—H13C	109.5	C31—C30—H30A	120.1
H13B—C13—H13C	109.5	C30—C31—C26	120.35 (17)
C7—C14—C15	113.73 (15)	C30—C31—H31A	119.8
C7—C14—H14A	108.8	C26—C31—H31A	119.8

### Hydrogen-bond geometry (Å, °)

**Table:**

*D*—H···*A*	*D*—H	H···*A*	*D*···*A*	*D*—H···*A*
N1—H1···O3^i^	0.87 (2)	2.09 (2)	2.9334 (18)	163.4 (18)
N2—H2···O3^i^	0.89 (2)	2.00 (2)	2.8610 (17)	162.8 (17)

Symmetry codes: (i) −*x*+3/2, *y*, −*z*+3/2.
